# Mandatory Biological/Targeted Synthetic Disease-Modifying Antirheumatic Drugs Dose Reduction on Risk of Serious Infections in Patients with Rheumatoid Arthritis: A Nationwide Nested Case–Control Study

**DOI:** 10.3390/biomedicines13122891

**Published:** 2025-11-26

**Authors:** Der-Yuan Chen, Ching-Heng Lin, Hsin-Hua Chen, Yi-Ming Chen, Kuo-Tung Tang

**Affiliations:** 1 Ph.D. Program in Translational Medicine, National Chung Hsing University, Taichung 402, Taiwan; dychen1957@gmail.com (D.-Y.C.); shc5555@hotmail.com (H.-H.C.); blacklark@gmail.com (Y.-M.C.); 2 Rheumatology and Immunology Center, China Medical University Hospital, Taichung 404, Taiwan; 3 College of Medicine, China Medical University, Taichung 404, Taiwan; 4 Institute of Medicine, Chung Shan Medical University, Taichung 402, Taiwan; 5 Department of Medical Research, Taichung Veterans General Hospital, Taichung 407, Taiwan; epid@vghtc.gov.tw; 6 Division of General Medicine, Department of Medicine, Taichung Veterans General Hospital, Taichung 407, Taiwan; 7 Department of Industrial Engineering and Enterprise Information, Tunghai University, Taichung 407, Taiwan; 8 Faculty of Medicine, National Yang Ming Chiao Tung University, Taipei 112, Taiwan; 9 Division of Allergy, Immunology and Rheumatology, Taichung Veterans General Hospital, Taichung 407, Taiwan; 10 Division of Translational Medicine, Department of Medical Research, Taichung Veterans General Hospital, Taichung 407, Taiwan

**Keywords:** antirheumatic agents, drug tapering, infections, pneumonia, rheumatoid arthritis

## Abstract

**Background**: We aimed to investigate the risk for a serious infection in rheumatoid arthritis (RA) patients after tapering the dose of biological/targeted synthetic disease-modifying antirheumatic drugs (b/tsDMARDs). **Methods**: This nested case–control study investigated the risk for a serious infection in RA patients who underwent mandatory b/tsDMARDs dose reduction 2.5 years after starting therapy with a single b/tsDMARD in the National Health Insurance Research Database (NHIRD). Cases were those patients who developed a serious infection afterwards. Matched controls were selected from those patients who did not develop a serious infection. We used unconditional logistic regression to analyze the odds ratios (ORs) of b/tsDMARDs dose reduction and discontinuation between cases and controls. **Results**: RA patients underwent an average dose reduction of 60%. Among a total of 268 cases and 1072 controls, we did not observe a lower risk for a serious infection in those patients who tapered or discontinued b/tsDMARDs. However, those patients who had discontinued b/tsDMARDs had a higher risk for a serious infection when compared with those who had not and had reduced their b/stDMARDs dose reduction below the average (i.e., ≤60%), with an adjusted OR of 1.48 (95%CI: 1.05, 2.09). **Conclusions**: Dose reduction in b/tsDMARDs in RA patients might not be associated with a lower risk for serious infection. Discontinuation of b/tsDMARDs, however, was likely associated with a higher risk for serious infection.

## 1. Introduction

Rheumatoid arthritis (RA) is an inflammatory arthritis characterized by chronic erosive synovitis resulting in disability [1]. The use of biologic/targeted synthetic disease-modifying antirheumatic drugs (b/tsDMARDs) is remarkably effective in treating RA, despite higher risks of infection [2,3]. For patients who have achieved remission or low disease activity (LDA), dose reduction in b/tsDMARDs is typically considered to alleviate economic burdens and adverse effects [4,5]. The European Alliance of Associations for Rheumatology (EULAR) recommends for patients in remission, tapering biologics after first reducing the dosage of glucocorticoids [6]. The impact of tapering or discontinuation of b/tsDMARDs remains to be determined [7,8]. For example, risks for serious infection are similar between those who tapered and had not tapered bDMARDs in randomized controlled trials, although these trials did not have sufficient power for such an outcome [8,9,10,11]. In dose-ranging trials of b/tsDMARDs, the infection risk appeared higher with the increase in the dosage [12,13]. Benefits of b/tsDMARDs dose reduction on safety profile, particularly in serious infections, remain to be explored. Given the limited data regarding the influence of b/tsDMARDs dose reduction on the risk for serious infection in RA patients, this issue is worth investigating.

The Taiwan Health Insurance Administration has put forward a dose-reducing policy for b/tsDMARDs since April 2013, enforcing a half dose reduction (dose-halving) and even discontinuation (a year later) from a standard dose for patients achieving remission or LDA after >2 years of a single bDMARD or tsDMARD [14]. At the same time, the standard dose can be resumed if the patient experiences a disease flare after certification by the National Health Insurance. It is assumed, therefore, these RA patients could be separated into two groups after around 2.5 years of a single bDMARD or tsDMARD: one group under reduced dose of b/tsDMARD and the other group under standard dose of b/tsDMARD. The mandatory governmental policy makes it an opportunity to examine the real-world impact of b/tsDMARDs dose reduction on the risk for serious infection in RA patients.

In this nationwide nested case–control study, we aimed to investigate the impact of b/tsDMARDs dose reduction on the risk of a serious infection in RA patients.

## 2. Materials and Methods

### 2.1. Patients

This nested case–control study was based on national longitudinal cohort data obtained from the National Health Insurance Research Database (NHIRD) [15], which contains comprehensive healthcare claims data from more than 99% of the Taiwanese population. Diseases in the NHIRD are coded according to the International Classifications of Diseases, 9th Revision, Clinical Modification (ICD-9-CM) codes for the period prior to 2016 and according to the ICD-10-CM codes thereafter [16]. Data in the NHIRD are de-identified before analysis. The need for informed consent from individuals was therefore waived for this study.

In the NHIRD covering a period from January 2000 to December 2017, we identified 13733 RA patients registered in the Catastrophic Illness Patients Database (CIPD). These patients must fulfill the 1987 revised criteria of the American College of Rheumatology (ACR) [17] (before August 2010) or the 2010 classification criteria of ACR/EULAR collaborative initiative for RA (after August 2010) [18]. This requirement was certified by two experienced rheumatologists before a patient was enrolled in the CIPD. All patients had received b/tsDMARDs according to the guidelines of the British Society for Rheumatology [19]. To investigate effect of the dose-reducing policy first implemented in April 2013, we included those patients who took a single b/tsDMARD while suffering from no serious infection for at least 2.5 years. This study was approved by the Institutional Review Board of Taichung Veterans General Hospital (IRB No. CE17178A), and in compliance with the Declaration of Helsinki.

### 2.2. Definitions

Serious infections included bacterial and opportunistic infections. Bacterial infections specifically included pneumonia, urinary tract infection, and septic arthritis which requires hospitalization or intravenous antibiotics. Opportunistic infections included herpes zoster which required oral/intravenous acyclovir or valacyclovir, cryptococcosis which required use of fluconazole, amphotericin, or flucytosine for ≥7 days, tuberculosis with ≥2 anti-tuberculosis drugs (isoniazid, rifampin, pyrazinamide, ethambutol, and/or moxifloxacin) for ≥7 days, and invasive aspergillosis which required the use of voriconazole, posaconazole, or amphotericin for ≥7 days. Details of the definitions are shown in Table S1. The follow-up period was the time period of 2.5 years after the start of b/tsDMARD use.

### 2.3. Cases and Controls

RA patients who had developed a serious infection at least 2.5 years after the start of b/tsDMARD therapy were included as cases. The index date of a serious infection was the time of its first documentation. Eligible controls were randomly selected (based on sorting by the random number; without replacement) from those RA patients who did not develop a serious infection for 2.5 years after starting b/tsDMARD therapy. Controls were matched to cases in a 1:4 ratio according to the follow-up periods and index dates. The average dose of b/tsDMARDs within 3 months of the index date was also calculated to represent the resulting dose after mandatory dose reduction. The percentage of dose reduction was calculated based on the defined daily dose (DDD) (Table S2).

### 2.4. Statistical Analysis

Results were presented as mean ± SD unless specified otherwise. Numerical variables were compared using Student’s *t*-test. Categorical variables were compared using the Chi-squared test. After the selection of potential variables in the multivariate analysis, we used unconditional logistic regression between cases and controls to determine the odds ratios (ORs) of b/tsDMARDs dose reduction and discontinuation. In an exploratory analysis in terms of the cut-off point of comparison, we used the average percentage in dose reduction in b/tsDMARDs, or 60%. In the stratification analysis, subgroup analyses were made between RA patients who used and had not used corticosteroids within 6 months prior to the index date. In the sensitivity analysis, we restricted the study population to patients who were diagnosed with RA from April 2011 (2 years before the dose-reducing policy) to December 2017. The analyses were completed using SAS software version 9.4 (SAS Institute Inc., Cary, NC, USA). A two-sided *p* value of <0.05 was considered statistically significant.

## 3. Results

### 3.1. Patients

**Figure 1:**
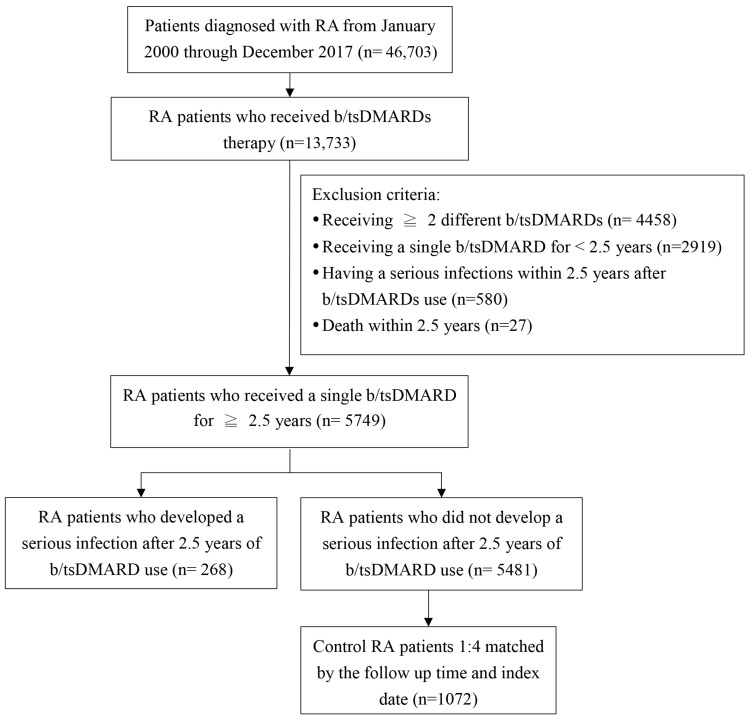
Study flow chart. b/tsDMARDs, biologic/targeted synthetic disease-modifying antirheumatic drugs; RA, rheumatoid arthritis.

Figure 1 shows a flowchart of patient enrollment. We identified a total of 5749 RA patients who had received therapy for at least 2.5 years with either a single bDMARD or a single tsDMARD. The average follow-up period of these patients was 3.7 years (for 2.5 years after starting b/tsDMARD). In the period, 268 (4.7%) patients developed a serious infection (1.3 events per 100 person-years). Half of them contracted pneumonia, and 1/4 of them contracted a urinary tract infection. To be noted, 1/10 of them contracted tuberculosis. We also selected 1072 matched RA controls without a serious infection. Baseline characteristics of the enrolled patients are shown in Table 1. Most of them were female and aged ≥50 years old. In terms of b/tsDMARDs, the majority of them underwent dose reduction and even discontinuation in some instances. On average, the RA patients underwent a dose reduction by ≤60%. Among these patients, 825 (62%) received therapy with etanercept, 355 (26%) with adalimumab, 54 (4%) with golimumab, 45 (3%) with abatacept, 35 (3%) with tocilizumab, and 26 (2%) with tofacitinib.

### 3.2. Risk Factors Associated with a Serious Infection in Those Receiving b/tsDMARDs

As shown in Table 2, we did not observe a change in risk for a serious infection in RA patients undergoing dose reduction or discontinuation when compared with those without such reductions. Notably, in the exploratory analysis, RA patients who had discontinued b/tsDMARDs showed a higher risk for a serious infection when compared with those who did not undergo b/stDMARDs dose reduction and those who underwent b/stDMARDs dose reduction by ≤60%, with an adjusted OR of 1.48 (95%CI: 1.05, 2.09).

### 3.3. Stratification Analysis

As shown in Table 3, the risk for a serious infection of RA patients appeared higher in the subgroup using corticosteroids. Among these patients, those who had discontinued b/tsDMARDs showed a trend toward a higher risk for a serious infection when compared with those who did not reduce the dose of b/tsDMARDs, with an adjusted OR of 2.99 (95%CI: 0.99, 9.09). Also, those who had discontinued b/tsDMARDs showed a higher risk for a serious infection when compared with those who underwent b/stDMARDs dose reduction by ≤60%, with an adjusted OR of 1.69 (95%CI: 1.10, 2.58).

### 3.4. Sensitivity Analysis

As shown in Table S3, the risk for a serious infection of RA patients appeared negatively associated with b/tsDMARDs dose reduction. Despite a lack of statistical significance, those who had discontinued b/tsDMARDs showed a higher risk for a serious infection when compared with those who did not undergo b/tsDMARDs dose reduction and those who underwent b/stDMARDs dose reduction by ≤60%, with an adjusted OR of 1.46 (95%CI: 0.42, 5.11) and 1.59 (95%CI: 0.76, 3.35).

## 4. Discussion

A serious infection is a significant complication in RA patients, especially for those patients receiving b/tsDMARDs. Dose reduction in b/tsDMARDs is recommended in the EULAR guideline [20]. Our results did not detect that patients who had dose reduction in b/tsDMARDs were associated with a lower risk of a serious infection. On the contrary, RA patients who discontinued b/tsDMARDs were associated with a 1.7 fold higher risk for a serious infection when compared with those who underwent b/tsDMARDs dose reduction by ≤60%.

Previous studies on RA patients reported 1.5- to 2-fold higher risk for a serious infection in b/tsDMARDs users when compared with csDMARDs users [2,21]. In the present study, the incidence rate of a serious infection was 1.3 per 100 person-years in RA patients from 2.5 years after they started using b/tsDMARDs. The low incidence rate was compatible with the previous observation of a decreased infection risk with time in these patients [22]. The benefit and harm of dose reduction or discontinuation of b/tsDMARDs remains controversial in recent decades. Potential benefits include lower costs and fewer side effects (e.g., serious infection). The potential harm is a disease flare. The PRESERVE trial showed no significant difference in disease activity after tapering an etanercept dose to half for 52 weeks [8]. In another DRESS trial, dose reduction in adalimumab or etanercept was non-inferior to usual care in terms of the proportion of patients developing a major flare within 18 months [9]. Similar findings were reported in RA patients receiving certolizumab or abatacept [10,11]. On the contrary, the Rheumatoid Arthritis in Ongoing Remission (RETRO) trial showed a higher risk for a relapse after the tapering or discontinuation of csDMARDs and bDMARDs [23]. The recent rheumatoid arthritis medication tapering (RHEUMTAP) cohort also showed that RA patients who reduced or stopped b/tsDMARDs were more likely to experience a disease flare at 2 years [24]. Nonetheless, none of the trials showed a lower risk for serious infection in those patients with tapered bDMARDs, despite insufficient power of these trials for the outcome. In contrast, we found a numerically higher risk for a serious infection in RA patients who had tapered or discontinued b/tsDMARDs. The exact mechanism is completely unknown. A possible explanation for the discrepancy in our results with the literature is the potentially increased disease activity leading to deteriorated functional status after mandatory b/tsDMARDs tapering and discontinuation. Both a high disease activity and a reduced physical function are known to be associated with a higher risk for a serious infection [22]. Moreover, sustained remission or LDA is associated with a lower risk for a serious infection in the CORRONA registry [25]. Another possibility is the laxity in preventive measures against various infectious diseases after b/tsDMARDs discontinuation. In our RA patients who used corticosteroids, the observed effects on the occurrence of a serious infection had become exaggerated. This may well reflect the higher disease activity in these patients or the added infection risk with concomitant use of corticosteroids [22].

Moreover, in the exploratory analysis, we found an even higher risk for a serious infection in those RA patients who had discontinued b/tsDMARDs when compared with those who reduced <60% dose of b/tsDMARDs. In addition, previous studies indicated that discontinuation of bDMARDs carries a significant risk for disease flares in most patients [20,23]. Moreover, in a randomized double-blinded trial of RA patients at remission under combination therapy of etanercept and methotrexate, remission was maintained in significantly more patients who discontinued methotrexate than in those who discontinued etanercept [26]. The 2021 ACR guideline of RA favors tapering csDMARDs over b/tsDMARDs in patients with remission or LDA for ≥6 months [27]. Our findings corroborate these results. In the RETRO trial, interestingly, 0 (0%) of 93 patients who tapered csDMARDs and b/tsDMARD, and 2 (2%) of 96 patients who discontinued all DMARDs developed infections [23]. The cost saving in RA patients should be balanced between their increased disease flares and possible increased infection risk in the attempt to discontinue b/tsDMARDs. Notwithstanding our findings, larger studies are required before a conclusion is made.

There are some limitations of our study. First, our study is a nested case–control study based on administrative data. It is associated with reduced precision and power due to sampling of controls when compared with the full cohort study [28]. Some serious infections, particularly herpes zoster, may not have been recorded. Reporting bias and reverse causation are possible. The causal relationship is difficult to establish. Second, the sample size is relatively small, especially for certain infections, to investigate the risk factors for a serious infection, despite our use of the national cohort. Due to that, we could not match cases and controls by age and sex. Assuming the proportion difference in b/tsDMARDs reduction between cases and controls equals 8.5% (as it is in our data), power of 0.8, and the type I error of 0.05, 310 case patients and 1240 control patients are required to be able to reject the null hypothesis. Third, we lack data in terms of disease activity of RA in the claims database, which is an unmeasured confounder. In fact, RA patients undergoing mandatory b/tsDMARDs dose reduction and discontinuation are likely to have a mildly increased disease activity although they were allowed to resume the standard dose if they experienced a disease flare. The end result might be an increased risk for a serious infection. Fourth, RA patients may undergo b/tsDMARDs dose reduction or discontinuation due to reasons other than the governmental policy, such as comorbidities. The proportion of methotrexate use was lower and comorbidities, such as chronic renal disease and malignancy, was higher in patients who discontinued b/tsDMARDs (Table S4). Nevertheless, we adjusted for these variables in the multivariate analysis, although residual confounding might exist. Fifth, our study was conducted in RA patients receiving a single b/tsDMARD for at least 2 years. This introduced the selection bias towards patients with better treatment responses and/or tolerability. Sixth, the smoking status of patients was not documented in the database despite its potential contribution to respiratory infections. Last, our patients were Han Chinese and the majority of patients received TNF-α antagonist; therefore, our findings may not be directly extrapolated to other countries or ethnicities and users of other b/tsDMARDs. Prospective international multicenter studies on a variety of b/tsDMARDs are needed to strengthen our findings.

## 5. Conclusions

In conclusion, dose reduction in b/tsDMARDs might not be associated with a lower risk for a serious infection. Moreover, discontinuation of b/tsDMARDs may be associated with an increased risk for a serious infection. The balance of benefit and risk should be considered before b/tsDMARD tapering and discontinuation. The decision to discontinue b/tsDMARDs should be cautiously made.

## Figures and Tables

**Table 1:** Characteristics of study subjects.

Characteristics	RA Patients with a Serious Infection (n = 268)	RA Patients Without a Serious Infection (n = 1072)	*p* Value
Age (years), n (%)			<0.001
20–39	14 (5.2)	169 (15.8)	
40–49	29 (10.8)	244 (22.8)	
50–59	81 (30.2)	384 (35.8)	
60–69	79 (29.5)	198 (18.5)	
≥70	65 (24.3)	77 (7.2)	
Gender, n (%)			0.001
Female	189 (70.5)	853 (79.6)	
Male	79 (29.5)	219 (20.4)	
b/tsDMARDs, n (%)			0.010
Without reduction	9 (3.4)	45 (4.2)	
With reduction	164 (61.2)	747 (69.7)	
Discontinuation	95 (35.4)	280 (26.1)	
Comorbidities, n (%)			
Diabetes mellitus	45 (16.8)	101 (9.4)	0.001
Chronic lung disease	57 (21.3)	168 (15.7)	0.028
Chronic renal disease	34 (12.7)	70 (6.5)	0.001
Malignancy	33 (12.3)	40 (3.7)	<0.001
Other medications ^a^, n (%)			
Corticosteroids	192 (71.6)	589 (54.9)	<0.001
Methotrexate	147 (54.9)	703 (65.6)	0.001
Leflunomide	49 (18.3)	124 (11.6)	0.003
Azathioprine	6 (2.2)	13 (1.2)	0.204
Cyclosporine	8 (3)	29 (2.7)	0.803
Serious infection, n (%)			
Pneumonia	138 (51.5)	N.A.	
Urinary tract infection	68 (25.4)	N.A.	
Septic arthritis	34 (12.7)	N.A.	
Tuberculosis	28 (10.4)	N.A.	
Herpes zoster	22 (8.2)	N.A.	
Cryptococcosis	11 (4.1)	N.A.	
Aspergillosis	2 (0.7)	N.A.	

^a^ use within 6 months prior to the index date. b/tsDMARDs, biological/targeted synthetic disease-modifying antirheumatic drugs; N.A., not available; RA, rheumatoid arthritis.

**Table 2:** Multivariable analysis of factors associated with a serious infection in patients with rheumatoid arthritis.

Characteristic	Primary Analysis		Exploratory Analysis	
	aOR (95%CI)	*p* Value	aOR (95%CI)	*p* Value
b/tsDMARDs				
Without reduction	1.00			
With reduction	1.28 (0.59, 2.80)	0.530		
Discontinuation	1.83 (0.82, 4.10)	0.143		
Without reduction or dose reduction < 60%			1.00	
Dose reduction ≥ 60%			1.10 (0.74, 1.64)	0.649
Discontinuation			1.48 (1.05, 2.09)	0.025
Age (years)				
20–39	1.00		1.00	
40–49	1.23 (0.62, 2.42)	0.561	1.23 (0.62, 2.44)	0.551
50–59	2.13 (1.16, 3.93)	0.016	2.13 (1.15, 3.93)	0.016
60–69	4.08 (2.18, 7.64)	<0.001	4.09 (2.18, 7.65)	<0.001
≥70	8.80 (4.50, 17.21)	<0.001	8.85 (4.52, 17.34)	<0.001
Male gender	1.66 (1.19, 2.29)	0.003	1.65 (1.19, 2.28)	0.003
Comorbidity				
Diabetes mellitus	1.20 (0.78, 1.85)	0.418	1.19 (0.77, 1.85)	0.426
Chronic lung disease	0.89 (0.61, 1.30)	0.552	0.88 (0.60, 1.29)	0.512
Chronic renal disease	1.36 (0.83, 2.23)	0.221	1.36 (0.83, 2.22)	0.225
Malignancy	2.95 (1.73, 5.01)	<0.001	2.93 (1.72, 4.99)	<0.001
Other medications ^a^				
Corticosteroids	1.96 (1.41, 2.70)	<0.001	1.97 (1.43, 2.71)	<0.001
Methotrexate	0.75 (0.55, 1.02)	0.070	0.75 (0.55, 1.02)	0.068
Leflunomide	1.49 (0.99, 2.26)	0.057	1.51 (1.00, 2.28)	0.052
Azathioprine	2.49 (0.86, 7.24)	0.094	2.49 (0.86, 7.21)	0.092
Cyclosporine	1.00 (0.42, 2.42)	0.996	1.01 (0.42, 2.44)	0.981

^a^ use within 6 months prior to the index date. aOR, adjusted odds ratio; b/tsDMARDs, biological/targeted synthetic disease-modifying antirheumatic drugs; CI, confidence interval.

**Table 3:** Multivariable analysis of factors associated with a serious infection, stratified by corticosteroids use ^a^.

Characteristic	Without Corticosteroids Use		With Corticosteroids Use	
	aOR	*p* Value	aOR	*p* Value
b/tsDMARDs				
Without reduction	1.00		1.00	
With reduction	1.07 (0.32, 3.59)	0.910	1.84 (0.63, 5.39)	0.264
Discontinuation	1.15 (0.33, 3.97)	0.824	2.99 (0.99, 9.09)	0.053
Without reduction or dose reduction < 60%	1.00		1.00	
Dose reduction ≥ 60%	1.32 (0.64, 2.71)	0.450	1.05 (0.64, 1.71)	0.860
Discontinuation	1.18 (0.62, 2,24)	0.619	1.69 (1.10, 2.58)	0.016

^a^ Adjusted for age, sex, comorbidities (diabetes mellitus, chronic lung disease, chronic renal disease, and malignancy), and use of other medications (methotrexate, leflunomide, azathioprine, and cyclosporine). aOR, adjusted odds ratio; b/tsDMARDs, biological/targeted synthetic disease-modifying antirheumatic drugs.

## Data Availability

The data that support the findings of this study are available on request from the corresponding author. The data are not publicly available due to privacy.

## Supplementary Materials

The following supporting information can be downloaded at: https://www.mdpi.com/article/10.3390/biomedicines13122891/s1, Table S1: Disease codes in the study; Table S2: Defined daily dose of biological/targeted synthetic disease-modifying antirheumatic drugs in the study; Table S3: Multivariable analysis of factors associated with a serious infection, in RA patients who received b/tsDMARDs from April 2011 to December 2017; Table S4: Characteristics of study subjects with and without b/tsDMARDs dose reduction.

## Abbreviations

The following abbreviations are used in this manuscript:

ACR	American College of Rheumatology
b/tsDMARDs	Biologic/targeted synthetic disease-modifying antirheumatic drugs
CIPD	Catastrophic Illness Patients Database
DDD	Defined daily dose
EULAR	European Alliance of Associations for Rheumatology
ICD-9-CM	International Classifications of Diseases, 9th Revision, Clinical Modification
LDA	Low disease activity
NHIRD	National Health Insurance Research Database
OR	Odds ratio
RA	Rheumatoid arthritis
