# Supplementary material for: Mandatory Biological/Targeted Synthetic Disease-Modifying Antirheumatic Drugs Dose Reduction on Risk of Serious Infections in Patients with Rheumatoid Arthritis: A Nationwide Nested Case–Control Study
Source: Biomedicines. 2025 Nov 26;13(12):2891. doi: 10.3390/biomedicines13122891 (PMC12730782; doi:10.3390/biomedicines13122891)
Supplement: Supplementary file 1 [file biomedicines-13-02891-s001.zip › biomedicines-3925946-supplementary.pdf]

Table S1. Disease codes in the study.

**Disease codes**	ICD-9 CM codes	ICD-10 CM codes
Rheumatoid arthritis	714.0	M05.x, M06.x
**Serious infection**		
Penumonia	480.x, 481, 482.x, 483.x, 484.x, 485, 486, 487.0, 487.1, 510.x, 513.0	J09, J10.0, J10.1, J11.0, J12-J18, J22, J85.0, J85.1, J85.2, J86.x, B25.0, A37.x
Urinary tract infection	590.0x, 590.1x, 590.2, 590.8x, 590.9, 595.0, 595.4, 595.89, 595.9, 597.x, 599.0	N11.0, N11.8, N11.9, N10, N15.1, N15.9, N12, N13.6, N16.0, N30.0x, N30.8x, N30.9, N34.0, N34.3, N39.0
Septic arthritis	711	M00.x, M01.x
Herpes zoster	053.x	B02.x
Cryptococcosis	117.5, 321.0	B45.x
Tuberculosis	010-018	A15-A19
Aspergillosis	117.3, 484.6	B44.x
**Comorbidity**		
Chronic lung disease	491.x, 492.x, 493.x, 494.x, 496, 517.x	J41-J47, J99.x
Chronic renal disease	581.x, 582.x, 583.x, 585.x-589.x, 593.9, 403.x-404.x, 250.4x, 274.1x, 447.3x, 440.1x, 572.4x, 753.10, 753.12, 753.13, 753.14, 753.15, 753.16, 753.17, 753.19, 403.01, 446.21	N00-N08, N27.x, I12, I13, M10.3, I77.3, E11.2x, E13.2x, E10.2x, I70.1. K76.7, Q60.x, Q61.1, Q61.2, Q61.3, Q61.4, Q61.5, Q61.8, Q61.9, I12.x, M31.0, N18.x, N19.x, N14.x, N15.0, N15.8, N26, N25
Diabetes mellitus	250.x	E10-E14

Table S2. Defined daily dose of biological/targeted synthetic disease-modifying antirheumatic drugs in the study.

ATC code	Drug name	Route	Defined Daily Dose (DDD) (mg)
L04AB01	etanercept	subcutaneous	7
L04AB04	adalimumab	subcutaneous	2.9
L04AB06	golimumab	subcutaneous	1.66
L04AB05	certolizumab pegol	subcutaneous	14
L04AC07	tocilizumab	subcutaneous	23
L04AC07	tocilizumab	intravenous	17
L04AA24	abatacept	subcutaneous	18
L04AA24	abatacept	intravenous	27
L04AA29	tofacitinib	oral	10

Table S3. Multivariable analysis of factors associated with a serious infection, in RA patients who received b/tsDMARDs from April 2011 to December 2017.

**Characteristic**	**aOR (95%CI)**	**p value**
**b/tsDMARDs**		
Without reduction	1.00	
With reduction	0.97 (0.33, 2.85)	0.953
Discontinuation	1.46 (0.42, 5.11)	0.550
Without reduction or dose reduction < 60%	1.00	
Dose reduction ≥ 60%	1.24 (0.64, 2.38)	0.523
Discontinuation	1.59 (0.76, 3,35)	0.222

aOR, adjusted odds ratio; b/tsDMARDs, biological/targeted synthetic disease-modifying antirheumatic drugs; CI, confidence interval; RA, rheumatoid arthritis.

Table S4. Characteristics of study subjects with and without b/tsDMARDs dose reduction.

**Characteristic**	**Without reduction (n=54)**	**With reduction (n=911)**	**Discontinuation (n=375)**	***P*-value**	**Without reduction or dose reduction < 60% (n=706)**	**Dose reduction ≥ 60% (n=259)**	**Discontinuation (n=375)**	***P*-value**
**Age (years), n (%)**				0.280				0.170
20-39	9[a]	128 (14.1)	130[a]		230[a]	48 (18.5)	130[a]	
40-49		189 (20.7)				48 (18.5)		
50-59	21 (38.9)	319 (35)	125 (33.3)		249 (35.3)	91 (35.1)	125 (33.3)	
60-69	16 (29.6)	184 (20.2)	77 (20.5)		146 (20.7)	54 (20.8)	77 (20.5)	
≥70	8 (14.8)	91 (10)	43 (11.5)		81 (11.5)	18 (6.9)	43 (11.5)	
**Gender, n (%)**				0.210				0.720
Female	37 (68.5)	708 (77.7)	297 (79.2)		544 (77.1)	201 (77.6)	297 (79.2)	
Male	17 (31.5)	203 (22.3)	78 (20.8)		162 (22.9)	58 (22.4)	78 (20.8)	
**Comorbidity, n (%)**								
Diabetes mellitus	10 (18.5)	95 (10.4)	41 (10.9)	0.179	80 (11.3)	25 (9.7)	41 (10.9)	0.759
Chronic lung disease	17 (31.5)	142 (15.6)	66 (17.6)	0.009	117 (16.6)	42 (16.2)	66 (17.6)	0.878
Chronic renal disease	7 (13)	57 (6.3)	40 (10.7)	0.009	51 (7.2)	13 (5)	40 (10.7)	0.024
Malignancy	29[a]		44 (11.7)	<0.001	15 (2.1)	14 (5.4)	44 (11.7)	<0.001

**Other medications, n (%)**								
Corticosteroids	29 (53.7)	554 (60.8)	198 (52.8)	0.024	435 (61.6)	148 (57.1)	198 (52.8)	0.018
Methotrexate	42 (77.8)	643 (70.6)	165 (44)	<0.001	505 (71.5)	180 (69.5)	165 (44)	<0.001
Leflunomide		128 (14.1)		0.099		28 (10.8)		0.153
Azathioprine	5[a]	13 (1.4)	55[a]	0.955	137[a]	3 (1.2)	55[a]	0.886
Cyclosporine		28 (3.1)		0.592		6 (2.3)		0.499

b/tsDMARDs, biological/targeted synthetic disease-modifying antirheumatic drugs; DDD, defined daily dose.

[a]Numbers of patients fewer than 3 are not specified on account of patient privacy according to National Health Insurance Research Database regulations.
